# Antimicrobial Sensitivity Pattern from Hospitalized Pneumonia Patients in National Referral Infectious Disease Hospital in Indonesia

**DOI:** 10.1155/2022/3455948

**Published:** 2022-08-29

**Authors:** Pompini Agustina Sitompul, Roza Indriani, Adria Rusli, Titi Sundari, Rosamarlina Rosamarlina, Teguh Sarry Hartono, Siti Maemun, Mohammad Syahril, Diar Riyanti Rudiatmoko, Vivi Setiawaty

**Affiliations:** ^1^Prof. Dr. Sulianti Saroso National Infectious Disease Hospital, Ministry of Health, Jakarta 14340, Indonesia; ^2^Faculty of Medicine, Universitas Indonesia, Jakarta 10430, Indonesia

## Abstract

**Background:**

Pneumonia is still a major global problem with high morbidity and mortality. The increasing number of pneumonia cases caused by bacteria, especially multidrug-resistant pathogens, increasing age of the population, patients with chronic disease (comorbid), and inappropriate antimicrobial therapy at initial administration make the treatment become less effective. These issues finally contribute to higher morbidity and mortality in cases of hospitalized pneumonia patients. Therefore, it is crucial to know the microbial pattern and select the therapy according to local antimicrobial sensitivity patterns.

**Method:**

A cross-sectional study was conducted for hospitalized pneumonia patients between January 2015 and December 2016 in Indonesia National Referral Infectious Disease Hospital. Data were collected from medical records to show patient characteristics, antimicrobial treatment data, culture examination, and bacterial sensitivity.

**Results:**

A total of 99 pneumonia patients required hospitalization and underwent sputum culture examination. The patients were mostly above 65 years old (32.3%) and male (57.6%). The most common comorbidities were pulmonary tuberculosis (21%), and the others were heart failure, chronic obstructive pulmonary disease (COPD), and HIV/AIDS. Based on the sputum culture, fungi were identified in most specimens (56%), while the bacteria identified were *Klebsiella pneumoniae* (14%), *Acinetobacter* sp. (12%), and *Pseudomonas* sp. (8%). Third-generation cephalosporin, such as ceftriaxone (50%), was commonly used as an antibiotic for pneumonia treatment.

**Conclusion:**

Most common bacteria isolated from sputum culture were *Klebsiella pneumoniae* which were more sensitive to the beta-lactam and aminoglycoside groups. The higher risk factors were age above 65 years old, being male, and having tuberculosis.

## 1. Introduction

Pneumonia is still the primary cause of hospitalization and death worldwide, especially in developing countries. In 2013, the Global Burden of Disease Study reported from 188 countries around the world that lower respiratory tract infection was the second highest cause of death [1, 2]. In Europe, mortality rates for community-acquired pneumonia (CAP) vary widely from country to country, ranging from <1% to 48%. [3] The cases of CAP increased with the increase in the patient's age. In the USA, the incidence was 24.8 cases per 10,000 adults annually and the highest proportion accounted for elderly aged between 65 and 79 years old (63.0 cases per 10,000 adults) and those older than 80 years (164.3 cases per 10,000 adults) [4].

The main factors associated with mortality in pneumonia are comorbid illness, such as neurologic diseases, the presence of a potential multidrug-resistant (MDR) pathogen, and very advanced age (>85 years) [5–7]. Chronic obstructive pulmonary disease (COPD) was the most frequent respiratory comorbidity in pneumonia cases, decreasing in frequency with age. Among the other relevant comorbidities, diabetes mellitus and chronic liver disease showed a similar decreasing trend with age, whereas cardiovascular, neurologic, and chronic renal failure increased significantly [5, 6].

*Streptococcus pneumoniae* was the most prevalent pathogen found in all age groups, regardless of the presence of comorbidity illness. Potential MDR pathogens such as *Staphylococcus aureus*, *Enterobacteriaceae*, and *Pseudomonas aeruginosa* were present in 9.1% of the cases diagnosed and occurred almost exclusively in patients with comorbidities [6, 8]. Based on recent study by El-Solh, patients with Legionnaires' disease had underlying comorbidities, 70% had steroid-dependent COPD, and 30% had myelodysplastic syndrome. Approximately 70% patients with *Staphylococcus aureus* and *H. influenzae* CAP had either underlying renal disease or COPD. *Pseudomonas aeruginosa* was identified in three patients, one from the CAP group with bronchiectasis caused by the history of pulmonary tuberculosis (TB), and two from the Nursing Home Acquired Pneumonia (NHAP) group, in which one had diabetes mellitus and the other had a history of hemiplegia secondary to cerebrovascular disease [5].

Although diagnosis of CAP etiological microorganisms is essential to determine appropriate antibiotic therapy, antimicrobial therapy should be empirically administered to avoid delaying treatment, which is associated with a significant mortality rate [9, 10]. The selection of empirical antibiotics must be adjusted to the local microorganism patterns and bacterial sensitivity to be more effective and not cause bacterial resistance [11].

Many antibiotics are no longer adequate for the treatment of pneumonia since bacterial resistance arises from improper and inappropriate use of empirical antibiotics, carried out by patients and health workers. The increasing resistance of pneumonia-causing bacteria to some antibiotics commonly used by clinicians in the initial administration of antibiotics causes a reduction in the effectiveness of treatment and higher morbidity and mortality. However, currently, there are no data regarding the pattern of microbial causes of pneumonia and antibiotic sensitivity in Indonesia. This study will investigate the most common etiological microorganism, its sensitivity to antibiotics, and the most common comorbid illnesses in pneumonia patients at the referral infectious disease hospital in Indonesia.

## 2. Materials and Methods

### 2.1. Study Design

This cross-sectional study collected data from the medical records of hospitalized pneumonia patients at Prof. Dr. Sulianti Saroso National Referral Infectious Disease Hospital during 2015-2016. This study obtained data based on the results of bacterial sensitivity tests to antibiotics, culture examination, and empirical antibiotic use in patients with hospitalized pneumonia.

### 2.2. Data Collection

All pneumonia patients hospitalized and diagnosed with ICD *X* code J18.9 between 2015 and 2016 were recruited. The total population used for this study was selected based on the inclusion criteria and exclusion criteria. The inclusion criteria were as follows: (1) patients aged >18 years diagnosed with pneumonia and hospitalized during 2015-2016, (2) received empirical therapy or antibiotics, (3) had a complete medical record including medical record number, age, gender, and treatment used, and (4) culture data and bacterial sensitivity tests were available. Incomplete medical records and patients who did not have sputum culture examination were excluded from this study.

### 2.3. Diagnosis of Pneumonia

Pneumonia was diagnosed based on the signs and symptoms of cough, purulent sputum changes, the presence of fever, or history of fever 38^o^C, chest pain, spasms, crackles, leukopenia (<4500/*μ*l), or leukocytosis (>10,000/*μ*l) with the addition of infiltrates or air bronchograms found on chest X-ray in posteroanterior view. Further examinations that can be done to diagnose pneumonia were culture examination or Gram staining test.

### 2.4. Microbiology Investigations

Sputum and endotracheal aspirate (ETA) samples were collected and sent to Clinical Microbiology Laboratory (CML) to be microscopically assessed for adequacy. Culture and identification were performed according to CML standard practice by plating on blood agar and MacConkey agar and biochemical reactions. Antibiotic susceptibility testing was performed using Mueller–Hinton agar and by standard disk diffusion procedures. Results were interpreted against the CLSI criteria for disk diffusion. The antibiotics susceptibility data were processed and analyzed using WHO-NET Version 5.6 program.

## 3. Results

This study was carried out based on patients' medical records. From a total of 289 hospitalized pneumonia patients, only 99 hospitalized patients diagnosed with pneumonia who fulfilled the inclusion and exclusion criteria for this study were included.

Based on Table 1, out of 99 pneumonia patients, the highest proportion included those over 65 years of age (32.3%). More than half of the patients were male (57.6%). The patients were dominated by high school graduates (56.6%). One-third of patients were housewives (34.3%), followed by private employees (28.3%).

The sample for culture examination was mostly obtained from sputum (89.9%). The culture examinations found a total of five pathogenic bacteria and fungi. Fungi were the most frequent microbiological agent accounted for 56%. *Klebsiella pneumonia* was the most prevalent (14%), followed by *Acinetobacter* sp. (12%), *Pseudomonas* sp. (8%), *Escherichia coli* (4%), and *Enterobacter* sp. (3%). Meanwhile, two samples had no microorganisms found during the culture examination (Table 2).

Empirical antibiotic therapy for patients treated in this hospital varied greatly. However, the most frequent empirical antibiotic therapies given were third-generation cephalosporins (ceftriaxone) at 49.5% and beta-lactam (meropenem) at 15.2% (Table 3).

The levels of bacterial sensitivity to antibiotics are shown in Table 4. *Klebsiella pneumonia* was highly sensitive to imipenem (100%), meropenem (100%), and amikacin (93%). Meanwhile, *Acinetobacter* sp. was more sensitive to amikacin (67%) and kanamycin (58%). Moreover, *Pseudomonas* sp. was more sensitive to levofloxacin (75%), amikacin (63%), and gentamicin (63%) compared with other antibiotics.

From 99 cases of hospitalized pneumonia, the patients had various comorbidities. The comorbidities are shown in Table 5, which are pulmonary TB (21%) which was the most prevalent comorbidity, heart failure (14%), human immunodeficiency virus infection and acquired immune deficiency syndrome (HIV/AIDS) (12%), and COPD (12%).

## 4. Discussion

Kosar et al. in Turkey found that out of 208 hospitalized pneumonia patients, the most comorbid diseases found were COPD, hypertension, and diabetes mellitus [12]. A study by Luna et al. in Argentina also found that the highest proportion of comorbid diseases in hospitalized pneumonia patients was COPD and diabetes mellitus [8]. Unlike two previous studies, this study found that the most prevalent comorbid illness in hospitalized pneumonia patient was TB. This might be due to the fact that Indonesia is ranked third for country with the highest number of TB cases in the world. Moreover, the national referral hospital for infectious diseases treated all of the infectious disease cases including TB and MDR TB cases.

Globally, *Streptococcus pneumonia* is known to be the most frequent cause of CAP. The diagnosis of etiology is growing with the discovery of pneumococcal urine antigen. The incidence of pneumonia cases is currently decreasing due to the pneumococcal vaccine, especially in developed countries [13]. Luna et al. stated that the most common germs found in pneumonia patients were *Streptococcus pneumonia*, *Staphylococcus* sp., atypical pathogens, anaerobic Gram-negative bacteria, and *Pseudomonas aeruginosa* [8]. Meanwhile, this study found that bacteria commonly found in sputum examination were *Klebsiella pneumoniae*. Rammaert et al.'s research in Cambodia stated that *Klebsiella pneumonia* was mostly found in pneumonia patients with female gender and diabetes mellitus as risk factors. The prevalence of high sequelae of TB in Cambodia was one of the factors that caused bronchiectasis colonization of *Klebsiella pneumonia* [14]. Samson et al. in Nigeria found that TB and bacterial infections occurred due to the immunocompromised status of TB patients and few TB patients also had comorbid infections caused by *Streptococcus pneumonia*, *Salmonella typhi*, or *Streptococcus milleri* [15].

Boonsarngsuk et al. found that the case of chronic *Klebsiella pneumonia* was characterized by a productive chronic cough, and in some cases, hemoptysis was found [16]. Chest X-ray of chronic *Klebsiella pneumonia* usually demonstrates consolidation which was mainly located at the apical and posterior segments of the right upper lobe and the superior segment of the right lower lobe. Due to zonal preferences in a chronic course, it may be mistaken for pulmonary tuberculosis [16]. From the study, there were two cases of chronic *Klebsiella pneumonia* that were mimicking pulmonary TB. This similar clinical condition is vital for the clinicians considering that many cases of *Klebsiella pneumonia* were diagnosed as pulmonary TB.

In this referral hospital, *Klebsiella pneumonia* had the highest sensitivity to the beta-lactam group antibiotics (imipenem and meropenem) and aminoglycoside group (amikacin). This was mainly due to the higher incidence of Gram-negative pneumonia and the administration of inappropriate antibiotics in the previous healthcare facilities, causing antibiotic resistance [17]. Administering antibiotics according to the sensitivity pattern at an early stage of the disease would reduce morbidity and mortality.

## 5. Conclusion

Hospitalized pneumonia patients were mostly above 65 years old and male with tuberculosis as the most frequent comorbidity in Indonesia. *Klebsiella pneumoniae* were the most common bacteria found in sputum culture and cephalosporin was frequently used as the empirical antibiotic. *Klebsiella pneumoniae* had higher sensitivity to the beta-lactam and aminoglycoside groups.

## Figures and Tables

**Table 1:** Characteristics of hospitalized patients with pneumonia.

Variable	N	Percentage
*Age (years)*		
18–25	6	6.1
26–35	12	12.1
36–45	9	9.1
46–55	18	18.2
56–65	22	22.2
>65	32	32.3
*Gender*		
Male	57	57.6
Female	42	42.4
*Educational status*		
Elementary school graduate	9	9.1
Junior high school graduate	10	10.1
Senior high school graduate	72	72.7
University graduate	5	5.1
Unknown	3	3.0
*Occupation*		
Housewife	34	34.3
Salesman	1	1.0
Workers	5	5.1
Students	1	1.0
Retired	3	3.0
Civil servants	5	5.1
Private employee	28	28.3
Entrepreneur	6	6.1
Unemployed	3	3.0
Unknown	10	10.1

**Table 2:** The results of culture examination in patients with pneumonia.

Material	N	Percentage
ETT	10	10.1
Sputum	89	89.9
*Results*		
*Acinetobacter* sp.	12	12.0
*Enterobacter* sp.	3	3.0
*Escherichia coli*	4	4.0
*Klebsiella pneumonia*	14	14.0
*Pseudomonas* sp.	8	8.0
None	2	3.0
Fungi	56	56

**Table 3:** The use of empirical antibiotics in pneumonia patients.

Empirical parenteral antimicrobial	N	Percentage
Cefepime	1	1.0
Cefoperazone	2	2.0
Cefoperazone/sulbactam	1	1.0
Cefotaxime	5	5.1
Ceftazidime	2	2.0
Ceftriaxone	49	49.5
Ciprofloxacin	2	2.0
Levofloxacin	8	8.1
Meropenem	15	15.2
Viccillin SX	2	2.0

**Table 4:** The use of empirical antibiotics in pneumonia patients.

Bacteria	Isolate	Amikacin (%)	Ciprofloxacin (%)	Gentamycin (%)	Imipenem (%)	Kanamycin (%)	Sulfa (%)	Tetramycin (%)	Levofloxacin (%)	Meropenem (%)	Ceftazidime (%)
*Klebsiella pneumoniae*	14	93	79	86	100	86	56	45	82	100	78
*Acinetobacter* sp.	12	67	42	50	44	58	50	20	36	45	50
*Pseudomonas* sp.	8	63	57	63	60		38	13	75	60	20

**Table 5:** Comorbid diseases in hospitalized pneumonia patients.

Comorbid disease	Percentage
Pulmonary TB	21
HIV	12
COPD	12
Stomatitis candidiasis	4
Toxoplasmosis	2
Hypertension	11
CVD/stroke	8
Chronic kidney disease	5
Heart failure	14

## Data Availability

The data used to support the findings of this study are available from the corresponding author upon request.
